# Homicidios en ciudades del sur de Sudamérica: desigualdades educativas y fluctuaciones económicas

**DOI:** 10.1590/0102-311XES228923

**Published:** 2024-06-21

**Authors:** Carlos Marcelo Leveau

**Affiliations:** 1 Instituto de Producción, Economía y Trabajo, Universidad Nacional de Lanús, Lanús, Argentina.; 2 Consejo Nacional de Investigaciones Científicas y Técnicas, Buenos Aires, Argentina.

**Keywords:** Homicidio, Recesión Económica, Factores Socioeconómicos, Área Urbana, Homicide, Economic Recession, Socioeconomic Factors, Urban Area, Homicídio, Recessão Econômica, Fatores Socioeconômicos, Área Urbana

## Abstract

Se sabe poco sobre cómo las fluctuaciones económicas afectan las desigualdades educativas en homicidios en países latinoamericanos. Los objetivos de este estudio fueron (a) analizar las variaciones temporales de las desigualdades relativas educacionales de la mortalidad por homicidio, y (b) comparar estas desigualdades entre años de crecimiento económico y años de recesión en ciudades del sur sudamericano durante el período 2000-2019. Se utilizaron datos de siete áreas urbanas, en tres países del Cono Sur Sudamericano: Mendoza y Rosario (Argentina); Belo Horizonte, Curitiba, Rio de Janeiro y São Paulo (Brasil); y Santiago (Chile). Se estimaron modelos de Poisson, utilizando como variables explicativas la edad, sexo, año, ciudad de residencia, año de expansión o recesión económica y nivel educativo. Encontramos diferencias marcadas en la evolución temporal de las tasas de homicidio entre las siete ciudades, aunque siempre las poblaciones de nivel educativo bajo fueron las más vulnerables. Las cuatro ciudades de Brasil, analizadas en conjunto, tuvieron desigualdades educativas relativas de homicidios mayores en años de recesión económica, con respecto a años de crecimiento económico. Por un lado, el uso de la fuerza indiscriminado por parte del Estado enfocado hacia grupos criminales parece haber llevado a una creciente desigualdad social de la mortalidad por homicidio. Por el otro, en un contexto de fragmentación criminal y crisis económica se podrían agravar estas desigualdades a través de mayores disputas territoriales entre grupos criminales.

## Abstract (en)

Information on how economic fluctuations affect educational inequalities in homicides in Latin America is scarce. This study aimed to: (a) analyze the temporal variations of educational inequalities related to homicide mortality and (b) compare these inequalities between years of economic growth and recession in southern South America cities from 2000 to 2019. Data from seven urban areas in three countries in the Southern Cone of South America were used: Mendoza and Rosario (Argentina); Belo Horizonte, Curitiba, Rio de Janeiro, and São Paulo (Brazil); and Santiago (Chile). Poisson models were estimated by using age, sex, city of residence, year of economic growth or recession, and schooling level as explanatory variables. Results showed significant differences in the temporal evolution of homicide rates in the seven cities, although populations with a low schooling level always showed the most vulnerability. The four Brazilian cities, analyzed together, showed greater educational inequalities related to homicides in years of economic recession when compared to those of economic growth. On the one hand, the indiscriminate use of force by the State against criminal groups seems to increase social inequality in homicide mortality. On the other hand, criminal fragmentation and economic crisis can exacerbate these inequalities by increasing territorial disputes between criminal groups.

## Resumo

São escassas as informações sobre como as flutuações econômicas afetam as desigualdades educacionais em homicídios na América Latina. Os objetivos deste estudo foram: (a) analisar as variações temporais das desigualdades educacionais relacionadas à mortalidade por homicídio, e (b) comparar essas desigualdades entre os anos de crescimento econômico e os anos de recessão nas cidades do sul da América do Sul no período de 2000 a 2019. Foram utilizados dados de sete áreas urbanas, em três países do Cone Sul da América do Sul: Mendoza e Rosário (Argentina); Belo Horizonte, Curitiba, Rio de Janeiro e São Paulo (Brasil); e Santiago (Chile). Os modelos de Poisson foram estimados utilizando como variáveis explicativas a idade, sexo, ano, cidade de residência, ano de expansão ou recessão econômica e nível de escolaridade. Os resultados mostraram diferenças significativas na evolução temporal das taxas de homicídio entre as sete cidades, apesar de que as populações com baixo nível de escolaridade sempre foram as mais vulneráveis. As quatro cidades brasileiras, analisadas em conjunto, apresentaram maiores desigualdades educacionais relacionadas a homicídios em anos de recessão econômica em relação aos anos de crescimento econômico. Por um lado, o uso indiscriminado da força pelo Estado contra grupos criminosos parece ter levado ao aumento da desigualdade social na mortalidade por homicídio. Por outro lado, em um contexto de fragmentação criminal e crise econômica, essas desigualdades podem ser exacerbadas pelo aumento das disputas territoriais entre grupos criminosos.

## Introducción

Comparada a otras regiones del planeta, la tasa de homicidio en la región de las Américas es la más alta ^1^. Además, mientras Europa ha logrado bajar a más de la mitad su tasa de homicidio durante las últimas dos décadas, en la región de las Américas las tasas de homicidio se han mantenido en los valores más altos durante el mismo periodo ^1^. Dentro de esta región, existe una heterogeneidad grande en las tasas de homicidio entre países y al interior de estos ^2^. Por ejemplo, entre los países sudamericanos, mientras Brasil, Colombia y Venezuela reportaron tasas entre 33 y 64 homicidios cada 100 mil habitantes durante 2017, entre los tres países del Cono Sur (Argentina, Chile y Uruguay) estas tasas fueron entre 4 y 9. Pero estas tasas nacionales enmascaran que las ciudades grandes son las más golpeadas por la violencia letal ^3^, impactando en mayor medida a poblaciones de nivel socioeconómico bajo ^4^ ^,^ ^5^ ^,^ ^6^.

Además de esta heterogeneidad geográfica, en los últimos años se han observado trayectorias disímiles de la violencia letal en ciudades latinoamericanas. En Brasil, durante 2000-2010, mega-ciudades como Rio de Janeiro y São Paulo disminuyeron sus tasas de homicidio, mientras varias ciudades costeras del norte aumentaron sus tasas ^7^ ^,^ ^8^. Aunque ha crecido la producción científica sobre homicidios en años recientes ^9^ y se ha hecho foco en las variaciones temporales recientes de las tasas de homicidio en algunas ciudades latinoamericanas, no se han analizado las variaciones temporales de las desigualdades sociales debido a estas muertes. En un contexto de descenso de la violencia letal, ¿cómo evolucionaron las desigualdades relativas sociales de la tasa de homicidio?

Una porción importante de las tasas elevadas de homicidio en las ciudades grandes latinoamericanas es explicada por el desenvolvimiento de los mercados ilícitos, especialmente el mercado de drogas ilegales ^10^. Por ejemplo, en Argentina se ha estimado que 4 de cada 10 homicidios estarían vinculados al narcotráfico ^11^. Desde finales del siglo XX, estas actividades económicas no solo han crecido como mercados de consumo en las mega-ciudades latinoamericanas a raíz de mejoras generales en el acceso a bienes de consumo ^12^, sino también en un contexto generalizado de baja institucionalidad, entendida como un debilitamiento del Estado, la familia, y otras instituciones en el marco del avance neoliberal sobre las sociedades latinoamericanas ^13^. De manera similar, se ha demostrado que aquellos países latinoamericanos con niveles bajos de institucionalidad, entendida como aquellas normas formales e informales que regulan las conductas violentas, registraban las tasas más altas de homicidio ^14^. Restringido al accionar directo del Estado, se ha planteado que la disminución de cohesión estatal, particularmente en las fuerzas de seguridad, estaría asociada al aumento de la violencia letal ^15^. Esta cohesión estatal implica tanto la regulación legal como ilegal del crimen organizado, y comprende la coordinación entre diferentes agencias de seguridad, entre diferentes niveles de gobierno (municipal, provincial, nacional), y estabilidad política y de mando de las agencias de seguridad ^15^.

Ahora bien, ¿cómo podría ser afectado este nivel de cohesión estatal por los ciclos económicos? Durante las últimas décadas, las sociedades latinoamericanas han sufrido crisis económicas recurrentes que han afectado de manera diferente a cada país. Recientemente, se ha encontrado que los años de crisis económica estuvieron asociados a aumentos de la tasa de homicidio en ciudades latinoamericanas ^16^. Durante periodos de recesión pueden crecer ciertas actividades económicas ilegales, como la venta de autopartes ligada al robo de vehículos, asociadas al aumento de la violencia letal ^17^. Con respecto al mercado de drogas ilegales, un incremento del desempleo estaría asociado a un aumento de vendedores de drogas, especialmente en áreas de nivel socioeconómico bajo ^18^, incrementando los niveles de competencia ^19^ en estas áreas. De esta manera, las crisis económicas podrían llevar a un aumento de la violencia letal debido a una mayor puja por el control de nuevos territorios. Además, es esperable que durante períodos de crisis económica aumenten los niveles de anomia ^20^, profundizando la disminución del control estatal de la violencia, especialmente a través de recortes presupuestarios relacionados a la aplicación de la ley ^19^. En este contexto, son las poblaciones de menor nivel socioeconómico las más vulnerables a este tipo de violencia.

Por lo tanto, planteamos la siguiente hipótesis: la regulación estatal del crimen organizado, tanto formal como informal, disminuye en periodos de crisis económica, llevando a un aumento de homicidios, mayor en poblaciones de nivel socioeconómico bajo con respecto a las poblaciones de mayor nivel socioeconómico. Los objetivos de este estudio son (a) analizar las variaciones temporales de las desigualdades relativas educacionales de la mortalidad por homicidio, y (b) comparar estas desigualdades entre años de crecimiento económico y años de recesión en ciudades del sur sudamericano durante el período 2000-2019.

## Métodos

Se utilizaron datos de siete áreas urbanas, en tres países del Cono Sur Sudamericano: Mendoza y Rosario (Argentina); Belo Horizonte, Curitiba, Rio de Janeiro y São Paulo (Brasil); y Santiago (Chile). Estas ciudades fueron seleccionadas con base en dos criterios: primero, por su gran tamaño poblacional (más de 1 millón de habitantes en 2020), lo que permitió tener una cantidad de homicidios anuales que permitieron estimar las desigualdades educativas en la mortalidad. En segundo lugar, en estas ciudades, durante el período 2000-2019, la proporción promedio de homicidios y muertes por lesión de intención no determinada que contenían información sobre el nivel educativo de los fallecidos fue del 80% o más. Salvo casos puntuales en Rosario (2000: 76%; 2014: 79%), Rio de Janeiro (2000-2001: 74%; 2002: 78%) y São Paulo (2006: 79%), todos los años tuvieron 80% o más de fallecidos con dato de nivel educativo (Material Suplementario - Tabla S1; https://cadernos.ensp.fiocruz.br/static/arquivo/supl-e00228923_8967.pdf). En Mendoza, Rosario, Belo Horizonte, y Santiago no parece haber un cambio temporal marcado en los porcentajes. En Curitiba, a partir de 2010, los porcentajes de fallecidos con nivel educativo rondaron el 98-99%. En Rio de Janeiro se observó una mejora gradual hasta 2009, pero luego los valores oscilaron entre 91-97%. En São Paulo, a partir de 2011, los porcentajes de fallecidos con nivel educativo comenzaron a superar el 90%, rondando entre 91-94%.

La definición de los límites de cada ciudad fue tanto la definición exacta que cada país brinda para sus áreas metropolitanas (ciudades de Brasil y Chile), como la combinación de departamentos que contienen los aglomerados urbanos definidos por el Instituto Nacional de Estadísticas y Censos (INDEC) de Argentina. En el caso de las ciudades de Brasil, fueron excluidos aquellos municipios con menos de 20 mil habitantes de acuerdo al censo de 2000. De esta manera, se excluyeron municipios periféricos que, en la mayoría de los casos, no comprendían la mancha urbana continua de las ciudades. Además, por su tamaño poblacional pequeño, una parte de estos municipios metropolitanos de menos de 20 mil habitantes fueron fusionados a otros municipios no metropolitanos en el IPUMS (Serie Integrada de Microdatos de Uso Público) ^21^, fuente de datos utilizada para obtener estimaciones poblaciones comparables por nivel educativo entre 2001 y 2010, como se verá más adelante. El Material Suplementario (Cuadro S1; https://cadernos.ensp.fiocruz.br/static//arquivo/supl-e00228923_8967.pdf) muestra el listado de municipios metropolitanos incluidos y excluidos en las ciudades de Brasil.

Se obtuvieron datos de homicidios (códigos de la Clasificación Internacional de Enfermedades - CIE-10: X85-X99) por edad, sexo (femenino/masculino), área de residencia (departamento en Argentina, municipio en Brasil y comuna en Chile) y nivel educativo para el período 2000-2019 del Ministerio de Salud de Argentina ^22^, Departamento de Informática del SUS (DATASUS) de Brasil ^23^ y del Ministerio de Salud de Chile ^24^ (en este último con datos de nivel educativo solo disponibles para 2000-2017). Según varias investigaciones en Argentina y Brasil ^25^ ^,^ ^26^ ^,^ ^27^ ^,^ ^28^ ^,^ ^29^ ^,^ ^30^, es necesario considerar las causas de muerte por lesión de intención no determinada cuando se estima la mortalidad por homicidio. Por lo tanto, en nuestro análisis se incluyeron las muertes con los códigos Y10-Y34 de la CIE-10. La edad se clasificó en tres grupos: 25-44, 45-64 y 65 o más años. El nivel educativo de los fallecidos fue utilizado como indicador del nivel socioeconómico debido a su disponibilidad en las estadísticas vitales de los tres países, práctica común en estudios similares comparando diferentes países ^31^ ^,^ ^32^. En las ciudades argentinas, los homicidios con dato de nivel educativo hasta secundario completo fueron categorizados como nivel educativo bajo, mientras que el nivel educativo medio-alto fue categorizado a partir de educación superior o universitaria incompleta. En las ciudades brasileras, en nivel educativo bajo fue considerado para defunciones con hasta 11 años de escolaridad, mientras que 12 o más años de escolaridad fueron categorizados como nivel educativo medio-alto. En Santiago, los homicidios de nivel educativo bajo fueron aquellos codificados con hasta nivel secundario, mientras que los homicidios con nivel educativo superior fueron categorizados como nivel educativo medio-alto. Debido al bajo número de homicidios de nivel educativo medio-alto en Mendoza y Rosario, una segunda categorización consideró este nivel como aquellas muertes con nivel educativo secundario completo o más, mientras que el nivel educativo bajo incluyó muertes hasta nivel secundario incompleto. Esta categorización solo fue utilizada en el segundo modelo de regresión (ver más adelante) y permitió estimar la tasa de homicidio por nivel educativo año a año en Mendoza y Rosario, como así también las variaciones temporales de la desigualdad relativa educativa por homicidio.

Para cada ciudad se utilizaron datos del producto interno bruto (PIB) per cápita estimados por Kummu et al. ^33^ para el periodo 2000-2015. Esta base de datos contiene el PIB per cápita subnacional anual (es decir, provincia en Argentina, estado en Brasil, y región en Chile) que asignamos a cada una de las siete ciudades. Los montos del PIB están en dólares estadounidenses de 2011 y se obtuvieron mediante la conversión de monedas locales a dólares utilizando paridades de poder adquisitivo (PPA) ^33^. Esta variable fue transformada en categórica indicando si el año fue de recesión (variación nula o negativa del PBI per cápita) o de crecimiento económico (variación positiva). En Mendoza y Rosario los años 2000-2002, 2009 y 2015 fueron de recesión. En Brasil, las cuatro ciudades tuvieron años de recesión en 2009, 2014 y 2015. Salvo Belo Horizonte, las restantes ciudades tuvieron otros años de recesión (Curitiba en 2005, Rio de Janeiro en 2001 y 2003; São Paulo durante 2001-2003). Por último, Santiago tuvo un año de recesión en 2009.

Para estimar tasas de homicidio y riesgos relativos de mortalidad, en los casos de Mendoza, Rosario, y Santiago se calcularon proyecciones poblacionales lineales utilizando datos censales (2001 y 2010 en Mendoza y Rosario, 2002 y 2017 en Santiago). En las ciudades de Brasil se utilizaron los datos procesados por IPUMS ^34^, debido a que en 2006 se pasó del sistema 8-3 (8 años de educación primaria y 3 años de educación secundaria) al sistema 9-3 (9 años de educación primaria y 3 años de educación secundaria). Ante este cambio, aquellas muertes clasificadas con 12 o más años de escolaridad pueden incluir fallecidos con secundario completo o con estudios terciarios o universitarios incompletos. Por este motivo, se utilizó la variable *EDATTAIN* de IPUMS cuyo objetivo es obtener estimaciones homogéneas de población por nivel educativo entre diferentes censos y países ^21^. En todas las ciudades se realizaron proyecciones poblacionales separadamente por sexo, grupo de edad y nivel educativo.

Utilizamos modelos de regresión de Poisson para probar la asociación entre nivel educativo y homicidio. Empleamos como variables explicativas la edad, el nivel educativo, el sexo y el año, mientras que los conteos agregados de homicidios y muertes por lesión de intención no determinada fueron la variable dependiente. Además, estimamos otros dos modelos. En el segundo modelo, incluimos una interacción entre el nivel educativo y el año para permitir una evolución diferencial en las tasas de homicidio y riesgos relativos de homicidio por nivel educativo. En el tercer modelo, incorporamos una interacción entre el nivel educativo y la variable categórica recesión/expansión para comparar tasas de homicidios y riesgos relativos de homicidio por nivel educativo, entre años de expansión económica y recesión, restringido al período 2000-2015. En los modelos segundo y tercero, se obtuvieron tasas marginales predichas para estimar las tasas de homicidio y riesgos relativos de homicidio por nivel educativo para cada año o entre años de recesión o expansión, respectivamente. Estos tres modelos se utilizaron en cada ciudad, de manera conjunta en las cuatro ciudades de Brasil y de manera conjunta en todas las ciudades. En el caso de los modelos con múltiples ciudades, se incluyó cada ciudad como efecto fijo. Debido a la presencia de colinealidad, la variable año fue excluida del tercer modelo, excepto en los análisis conjuntos de ciudades de Brasil y todas las ciudades. Como prueba de robustez de los resultados, recalculamos todos los modelos, considerando solo el recuento de homicidios como variable dependiente. Los datos se analizaron utilizando el programa Stata versión 13.1 (https://www.stata.com).

## Resultados

La Tabla 1 muestra las tasas brutas de homicidios y muertes por lesión de intención no determinada por año y ciudad. Si se considera un nivel bajo de mortalidad por muertes por lesión de intención no determinada, Santiago cuenta con la tasa promedio más baja de homicidio, mientras que Curitiba tuvo la tasa promedio de homicidio más elevada. Considerando el porcentaje de homicidios con respecto a las muertes por lesión de intención no determinada, en Mendoza se registró un aumento de las últimas, superando el 30% a partir de 2016 (Tabla 2). En Rosario, las muertes por lesión de intención no determinada superaron el 80% durante 2008-2013. Es notorio el salto en el número de homicidios entre 2013 y 2014, de 15 a 121 respectivamente (Tabla 3). En Belo Horizonte, Curitiba y São Paulo se observaron aumentos del porcentaje de muertes por lesión de intención no determinada en los últimos años del periodo 2000-2019. En paralelo, se observaron disminuciones en la cantidad de homicidios notificados en estas ciudades (Tabla 3). En Rio de Janeiro, las muertes por lesión de intención no determinada superaron el 40% durante el periodo 2007-2009, luego disminuyó este porcentaje para finalmente registrar un 72% en 2019 (Tabla 2). Entre 2018 y 2019, los homicidios pasaron de 1.941 a 1.051, mientras que las muertes por lesión de intención no determinada de 927 a 2.703 (Tabla 3). Por último, a excepción de los años 2000-2001 y 2004, en Santiago no se registraron muertes por lesión de intención no determinada durante 2000-2017.

**Tabla 1:** Tasas brutas de mortalidad, cada 100.000 habitantes, por homicidio y muerte por lesión de intención no determinada (MLIND-Y10-Y34) en siete ciudades de Sudamérica, 2000-2019.

Año	Mendoza		Rosario		Belo Horizonte		Curitiba		Rio de Janeiro		São Paulo		Santiago	
	Homicidios	MLIND	Homicidios	MLIND	Homicidios	MLIND	Homicidios	MLIND	Homicidios	MLIND	Homicidios	MLIND	Homicidios	MLIND
2000	13,9	0,4	5,6	0,0	28,4	9,3	20,7	8,1	36,0	10,4	53,0	11,4	6,3	2,7
2001	12,6	0,8	8,9	0,1	33,9	10,6	24,7	5,6	34,0	12,8	51,2	9,5	6,5	1,1
2002	15,9	0,9	6,5	0,5	38,1	5,9	27,5	6,9	40,0	12,4	45,7	16,0	6,5	0,0
2003	16,0	0,0	6,2	2,6	50,1	8,7	35,1	5,1	39,4	14,7	42,8	9,0	5,9	0,0
2004	8,2	0,3	4,0	2,5	55,7	10,2	35,3	2,5	39,8	11,1	34,1	11,3	6,9	0,7
2005	8,5	0,3	5,0	3,6	47,7	11,7	37,0	1,9	38,0	15,6	26,7	11,2	8,0	0,0
2006	8,9	0,6	3,9	5,6	43,3	14,0	39,7	3,0	39,6	12,8	23,1	7,2	7,5	0,0
2007	6,1	0,1	4,3	5,4	41,1	13,0	35,9	4,8	33,2	23,0	18,4	8,4	6,0	0,0
2008	6,4	0,1	2,3	12,1	35,8	12,4	45,6	4,4	27,5	25,2	17,3	7,5	5,5	0,0
2009	5,6	0,7	2,2	10,0	31,6	16,6	51,0	4,7	27,6	28,1	17,2	8,2	6,3	0,0
2010	4,4	0,6	1,8	18,5	30,7	11,0	50,5	3,7	30,1	11,9	15,5	8,2	5,6	0,0
2011	5,0	0,2	1,3	15,8	33,9	16,2	45,8	7,7	26,0	15,0	14,8	8,9	6,0	0,0
2012	5,1	0,4	1,1	11,4	32,1	8,0	40,4	7,7	24,3	13,6	16,3	6,8	4,9	0,0
2013	4,9	0,7	0,8	13,1	32,6	10,0	35,0	8,4	24,0	14,5	14,3	6,5	5,7	0,0
2014	5,7	1,0	6,0	4,9	30,7	9,6	36,5	8,7	24,6	7,3	13,8	6,8	7,4	0,0
2015	4,7	1,0	4,7	4,1	25,1	8,2	32,3	8,1	20,5	7,6	11,3	6,4	6,1	0,0
2016	3,6	1,7	4,1	5,4	25,4	9,8	32,8	4,8	22,0	10,3	9,3	6,6	5,5	0,0
2017	2,8	1,4	2,7	5,5	21,4	11,2	27,6	4,6	24,2	10,3	7,9	8,4	3,4	0,0
2018	2,7	1,2	4,4	4,6	17,2	8,8	23,1	3,4	22,0	10,5	6,1	15,8	ND	ND
2019	1,6	2,5	2,9	3,9	13,0	12,9	16,7	4,9	11,7	30,0	4,8	14,5	ND	ND
Tasa promedio	7,1	0,7	3,9	6,5	33,4	10,9	34,7	5,4	29,2	14,9	22,2	9,4	6,1	0,2

ND: datos no disponibles.

**Tabla 2:** Porcentajes de muertes por homicidio y muerte por lesión de intención no determinada (MLIND-Y10-Y34), con respecto al total de ambas muertes en cada año, en siete ciudades de Sudamérica, 2000-2019.

Año	Mendoza		Rosario		Belo Horizonte		Curitiba		Rio de Janeiro		São Paulo		Santiago	
	Homicidios	MLIND	Homicidios	MLIND	Homicidios	MLIND	Homicidios	MLIND	Homicidios	MLIND	Homicidios	MLIND	Homicidios	MLIND
2000	97,1	2,9	100,0	0,0	75,3	24,7	71,9	28,1	77,6	22,4	82,3	17,7	70,1	29,9
2001	94,4	5,6	98,5	1,5	76,2	23,8	81,5	18,5	72,6	27,4	84,4	15,6	86,0	14,0
2002	94,8	5,2	92,7	7,3	86,6	13,4	80,0	20,0	76,3	23,7	74,1	25,9	100,0	0,0
2003	100,0	0,0	70,3	29,7	85,3	14,7	87,4	12,6	72,9	27,1	82,6	17,4	100,0	0,0
2004	96,5	3,5	61,0	39,0	84,6	15,4	93,4	6,6	78,1	21,9	75,1	24,9	91,3	8,7
2005	96,9	3,1	58,3	41,7	80,3	19,7	95,2	4,8	70,9	29,1	70,5	29,5	100,0	0,0
2006	93,3	6,7	41,0	59,0	75,5	24,5	93,1	6,9	75,5	24,5	76,4	23,6	100,0	0,0
2007	98,1	1,9	44,1	55,9	76,0	24,0	88,3	11,7	59,1	40,9	68,8	31,2	100,0	0,0
2008	98,3	1,7	15,7	84,3	74,2	25,8	91,2	8,8	52,2	47,8	69,9	30,1	100,0	0,0
2009	88,9	11,1	18,2	81,8	65,6	34,4	91,5	8,5	49,6	50,4	67,7	32,3	100,0	0,0
2010	87,5	12,5	8,9	91,1	73,5	26,5	93,2	6,8	71,7	28,3	65,3	34,7	100,0	0,0
2011	96,7	3,3	7,4	92,6	67,7	32,3	85,6	14,4	63,4	36,6	62,6	37,4	100,0	0,0
2012	92,9	7,1	8,9	91,1	80,0	20,0	84,1	15,9	64,1	35,9	70,5	29,5	100,0	0,0
2013	87,2	12,8	5,8	94,2	76,6	23,4	80,6	19,4	62,3	37,7	68,6	31,4	100,0	0,0
2014	85,1	14,9	55,0	45,0	76,2	23,8	80,8	19,2	77,1	22,9	67,0	33,0	100,0	0,0
2015	81,9	18,1	53,6	46,4	75,4	24,6	80,0	20,0	73,0	27,0	63,9	36,1	100,0	0,0
2016	68,1	31,9	43,0	57,0	72,1	27,9	87,2	12,8	68,2	31,8	58,5	41,5	100,0	0,0
2017	66,7	33,3	32,4	67,6	65,8	34,2	85,8	14,2	70,2	29,8	48,6	51,4	100,0	0,0
2018	69,5	30,5	48,8	51,2	66,2	33,8	87,1	12,9	67,7	32,3	27,8	72,2	ND	ND
2019	39,4	60,6	42,6	57,4	50,2	49,8	77,2	22,8	28,0	72,0	24,9	75,1	ND	ND

ND: datos no disponibles.

**Tabla 3:** Muertes por homicidio y muerte por lesión de intención no determinada (MLIND-Y10-Y34) en siete ciudades de Sudamérica, 2000-2019.

Año	Mendoza		Rosario		Belo Horizonte		Curitiba		Rio de Janeiro		São Paulo		Santiago	
	Homicidios	MLIND	Homicidios	MLIND	Homicidios	MLIND	Homicidios	MLIND	Homicidios	MLIND	Homicidios	MLIND	Homicidios	MLIND
2000	68	2	37	0	592	194	284	111	2.249	648	5.066	1.089	220	94
2001	67	4	64	1	721	225	347	79	2.163	816	4.990	924	227	37
2002	91	5	51	4	831	129	395	99	2.590	805	4.542	1.589	230	0
2003	99	0	52	22	1.117	193	518	75	2.596	965	4.342	917	214	0
2004	55	2	36	23	1.272	232	534	38	2.664	746	3.537	1.174	251	24
2005	62	2	49	35	1.115	274	574	29	2.595	1.065	2.825	1.181	297	0
2006	70	5	41	59	1.038	336	633	47	2.747	892	2.504	774	282	0
2007	52	1	49	62	1.011	319	588	78	2.345	1.626	2.035	924	228	0
2008	59	1	28	150	903	314	767	74	1.982	1.813	1.959	845	214	0
2009	56	7	30	135	818	429	883	82	2.025	2.061	1.996	951	246	0
2010	49	7	27	278	816	294	906	66	2.258	891	1.852	985	222	0
2011	59	2	20	249	928	443	841	142	1.985	1.147	1.802	1.078	244	0
2012	65	5	19	195	903	226	765	145	1.889	1.057	2.029	849	202	0
2013	68	10	15	243	944	289	682	164	1.905	1.154	1.829	836	237	0
2014	86	15	121	99	914	285	736	175	1.994	593	1.809	891	314	0
2015	77	17	103	89	771	252	673	168	1.697	629	1.532	864	265	0
2016	64	30	96	127	803	311	707	104	1.861	869	1.287	912	241	0
2017	54	27	68	142	699	364	615	102	2.090	887	1.133	1.200	154	0
2018	57	25	123	129	581	297	534	79	1.941	927	893	2.319	ND	ND
2019	37	57	87	117	453	450	399	118	1.051	2.703	727	2.198	ND	ND
Total	1.295	224	1.116	2.159	17.230	5.856	12.381	1.975	42.627	22.294	48.689	22.500	4.288	155

ND: datos no disponibles.

**Figura 1:**
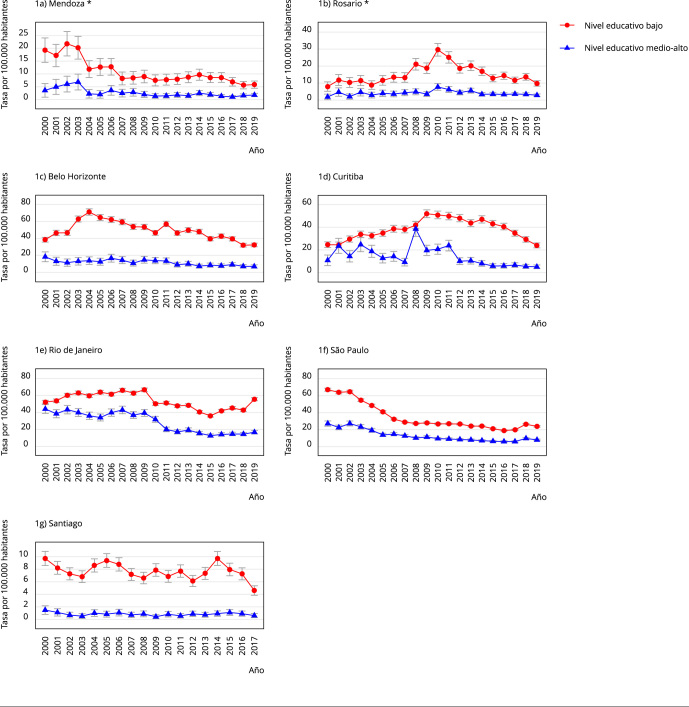
Tasa anual de homicidio según nivel educativo en siete ciudades de Sudamérica, 2000-2019.

La Figura 1 muestra las tasas de homicidio más muertes por lesión de intención no determinada, desagregadas por nivel educativo, y ajustadas por sexo y grupos de edad. En las siete ciudades, las tasas de homicidio en población de nivel educativo bajo fueron superiores a las tasas en población de nivel educativo medio-alto en casi todos los años (Figura 1). En Brasil, las cuatro ciudades muestran diferencias en la evolución de las tasas de homicidio por nivel educativo durante 2000-2019. En Belo Horizonte, las tasas de homicidio en población de nivel educativo bajo aumentaron hasta 2004 para luego descender en la mayoría de los años, mientras que las tasas de homicidio en población de nivel educativo medio-alto mostraron valores estables durante 2000-2010 y disminución durante 2011-2019 (Figura 1). En Curitiba, las tasas de homicidio en población de nivel educativo bajo aumentaron hasta 2009 para luego descender en la mayoría de los años, mientras que las tasas de homicidio en población de nivel educativo medio-alto descendieron a partir de 2012. En Rio de Janeiro, ambos niveles educativos parecen mostrar un descenso de la tasa de homicidio durante 2010-2015, para luego mostrar estabilidad (nivel educativo medio-alto) o aumento de la tasa de homicidio (nivel educativo bajo). En São Paulo, ambos niveles educativos muestran mayormente un descenso constante de la tasa de homicidio (Figura 1). En Argentina, Mendoza y Rosario, se mostraron diferencias en la variación temporal de las tasas de homicidio. En Mendoza, en ambos niveles educativos se observaron las tasas más altas de homicidio durante 2000-2003. En Rosario, las tasas de homicidio en población de nivel educativo bajo alcanzaron un pico en 2010, mientras que las tasas de homicidio en población de nivel educativo medio-alto se mantuvieron estables durante 2000-2019. Por último, Santiago (Chile) tuvo tres picos de la tasa de homicidio en población de nivel educativo bajo (2000, 2005 y 2014), mientras que las tasas de homicidio en población de nivel educativo medio-alto se mantuvieron estables.

Nota: las barras verticales son intervalos de 95% de confianza (IC95%). Tasas de mortalidad estimadas, incluyendo todas las variables explicativas y una interacción entre el año y el nivel educativo. La variable dependiente son conteos agregados de homicidios (X85-X99) y muerte por lesión de intención no determinada (Y10-Y34). * Nivel educativo bajo es hasta secundario incompleto, mientras que en el resto de las ciudades llega hasta secundario completo.

**Figura 2:**
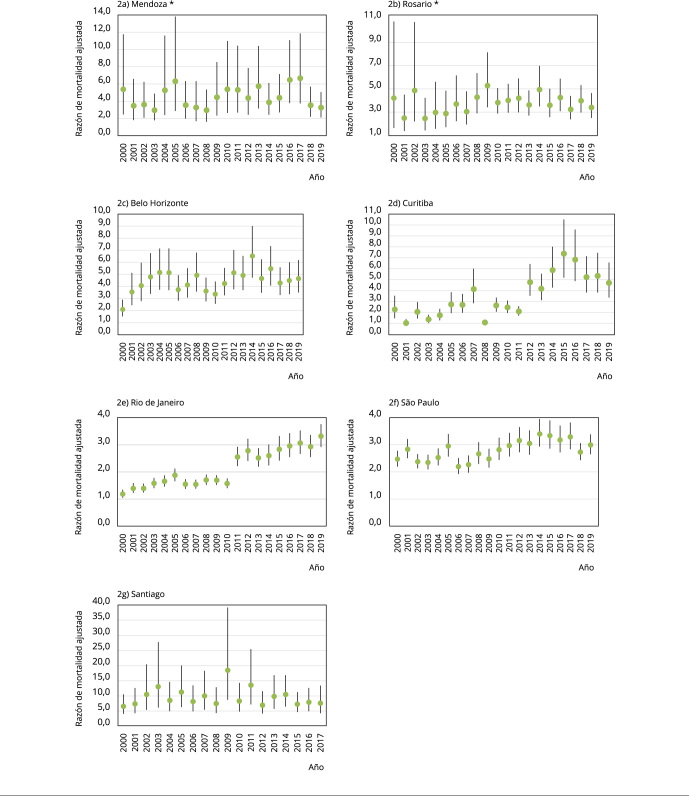
Razones de mortalidad anual, tomando como referencia la mortalidad por homicidio en población de nivel educativo medio-alto, en siete ciudades de Sudamérica, 2000-2019.

Las cuatro ciudades brasileras tuvieron desigualdades educacionales de homicidio menores comparadas a Mendoza y Rosario, y Santiago (Tabla 4). La Figura 2 muestra las desigualdades educativas relativas anuales en cada ciudad. Salvo Belo Horizonte, en Curitiba, Rio de Janeiro y São Paulo se observaron aumentos de la desigualdad relativa educacional por homicidios durante 2000-2019 (Figura 2). En Curitiba y Rio de Janeiro se observó un aumento importante de estas desigualdades a partir de 2012 y 2011, respectivamente. En Mendoza, Rosario y Santiago parecen observarse aumentos de las desigualdades educacionales del homicidio.

**Tabla 4:** Riesgos relativos (RR) de mortalidad para la población con nivel educativo bajo (utilizando como referencia la población con nivel educativo medio-alto) en siete ciudades de Sudamérica, 2000-2019.

País/Ciudad	RR * (IC95%)		
	Homicidios+MLIND	Homicidios
Argentina		
Mendoza	8,01 (6,38-10,04)	9,29 (7,15-12,08)
Rosario	7,38 (6,29-8,64)	18,21 (12,24-27,08)
Brasil		
Belo Horizonte	4,42 (4,13-4,72)	5,88 (5,37-6,43)
Curitiba	3,05 (2,86-3,25)	3,24 (3,02-3,47)
Rio de Janeiro	2,01 (1,95-2,06)	2,03 (1,96-2,10)
São Paulo	2,75 (2,67-2,83)	2,79 (2,69-2,89)
Chile		
Santiago **	9,01 (7,92-10,24)	9,49 (8,31-10,83)

IC95: intervalo de 95% de confianza; MLIND: muerte por lesión de intención no determinada (Y10-Y34).

Nota: todas las estimaciones provienen de modelos multivariados de Poisson en cada ciudad, ajustando por sexo, edad y año.

* Todos los riesgos relativos con valor de p < 0,001;

** Periodo 2000-2017.

Nota: las barras verticales son intervalos de 95% de confianza (IC95%) para las razones de tasas de mortalidad calculadas con la mortalidad, incluidas todas las variables explicativas y una interacción entre el año y el nivel educativo. La variable dependiente son conteos agregados de homicidios (X85-X99) y muerte por lesión de intención no determinada (Y10-Y34). * Nivel educativo bajo es hasta secundario incompleto, mientras que en el resto de las ciudades llega hasta secundario completo.

La Tabla 5 muestra las tasas de homicidio, de acuerdo al nivel educativo, y razones de mortalidad estimadas en años de crecimiento económico y recesión. En todas las ciudades, los riesgos relativos de desigualdad educativa por homicidio fueron mayores en años de recesión, comparados a años de expansión económica. El cociente entre ambos riesgos relativos, recesión con respecto expansión, mostró aumentos estadísticamente significativos (valor de p < 0,05) de la desigualdad educativa por homicidio en años de recesión en Curitiba, Rio de Janeiro, São Paulo, y las cuatro ciudades brasileras consideradas en conjunto (Tabla 6).

**Tabla 5:** Tasas de homicidio ajustadas y riesgos relativos (RR) entre grupos educativos (desigualdad relativa) en años de recesión *versus* años de expansión, 2000-2015.

País/Ciudad	Nivel educativo	Tasas de homicidio (IC95%)		RR (IC95%)	
		Expansión	Recesión	Expansión	Recesión
Argentina					
Mendoza	Medio-alto	1,01 (0,69-1,33)	1,12 (0,57-1,67)	Referencia	Referencia
	Bajo	8,32 (7,70-8,94)	11,09 (9,96-12,23)	8,22 (5,95-11,35)	9,88 (5,99-16,30)
Rosario	Medio-alto	2,24 (1,77-2,70)	1,14 (0,59-1,68)	Referencia	Referencia
	Bajo	15,61 (14,86-16,36)	11,36 (10,35-12,37)	6,98 (5,64-8,63)	10,01 (6,17-16,23)
Brasil					
Belo Horizonte	Medio-alto	12,64 (11,56-13,72)	9,61 (8,02-11,21)	Referencia	Referencia
	Bajo	53,66 (52,86-54,74)	46,35 (44,69-48,01)	4,24 (3,89-4,62)	4,82 (4,07-5,71)
Curitiba	Medio-alto	18,14 (16,76-19,53)	10,61 (9,05-12,17)	Referencia	Referencia
	Bajo	38,70 (39,73-37,67)	44,55 (42,87-46,22)	2,13 (1,97-2,31)	4,20 (3,61-4,88)
Rio de Janeiro	Medio-alto	30,94 (29,83-32,05)	25,72 (24,29-27,14)	Referencia	Referencia
	Bajo	55,30 (54,62-55,97)	50,29 (49,41-51,17)	1,79 (1,72-1,85)	1,96 (1,85-2,07)
São Paulo	Medio-alto	11,40 (10,91-11,89)	12,68 (12,03-13,34)	Referencia	Referencia
	Bajo	32,58 (32,17-32,99)	39,32 (38,76-39,87)	2,86 (2,74-2,98)	3,10 (2,94-3,27)
Chile					
Santiago	Medio-alto	0,89 (0,77-1,01)	0,43 (0,11-0,75)	Referencia	Referencia
	Bajo	7,90 (7,62-8,18)	7,89 (6,89-8,90)	8,88 (7,72-10,22)	18,41 (8,68-39,03)
Ciudades de Brasil *	Medio-alto	18,49 (18,02-18,97)	25,63 (24,69-26,68)	Referencia	Referencia
	Bajo	42,98 (42,55-43,40)	65,14 (63,75-66,54)	2,32 (2,27-2,38)	2,54 (2,45-2,64)
Todas las ciudades *	Medio-alto	11,42 (11,13-11,70)	15,28 (14,67-15,88)	Referencia	Referencia
	Bajo	29,25 (28,97-29,54)	40,67 (39,87-41,47)	2,56 (2,50-2,63)	2,66 (2,57-2,76)

IC95%: intervalo de 95% de confianza.

Nota: todas las estimaciones resultan de modelos de Poisson, cuya variable dependiente son conteos agregados de homicidios (X85-X99) y muerte por lesión de intención no determinada (Y10-Y34), ajustando por sexo y edad.

* Ambos modelos además incluyen año y ciudad como efectos fijos.

**Tabla 6:** Cociente entre riesgos relativos (RR) de desigualdad educativa de homicidio, tomando la desigualdad educativa de homicidio en años de expansión económica como referencia, 2000-2015.

País/Ciudad	Tipo de año	**Cociente de desigualdades educativas entre años de recesión *versus* expansión**
Argentina		
Mendoza	Expansión	Referencia
	Recesión	1,20 (0,66-2,18)
Rosario	Expansión	Referencia
	Recesión	1,43 (0,85-2,43)
Brasil		
Belo Horizonte	Expansión	Referencia
	Recesión	1,14 (0,94-1,37)
Curitiba	Expansión	Referencia
	Recesión	1,97 (1,66-2,33) *
Rio de Janeiro	Expansión	Referencia
	Recesión	1,09 (1,02-1,17) **
São Paulo	Expansión	Referencia
	Recesión	1,08 (1,01-1,16) **
Chile		
Santiago	Expansión	Referencia
	Recesión	2,07 (0,96-4,45) ***
Ciudades de Brasil ^#^	Expansión	Referencia
	Recesión	1,09 (1,05-1,14) *
Todas las ciudades ^#^	Expansión	Referencia
	Recesión	1,04 (0,99-1,09) ***

IC95%: intervalo de 95% de confianza.

Nota: todas las estimaciones resultan de modelos de Poisson, cuya variable dependiente son conteos agregados de homicidios (X85-X99) y muerte por lesión de intención no determinada (Y10-Y34), ajustando por sexo y edad.

* Valor de p < 0,001;

** Valor de p < 0,05;

*** Valor de p < 0,10.

^#^ Ambos modelos además incluyen año y ciudad como efectos fijos.

**Figura 3:**
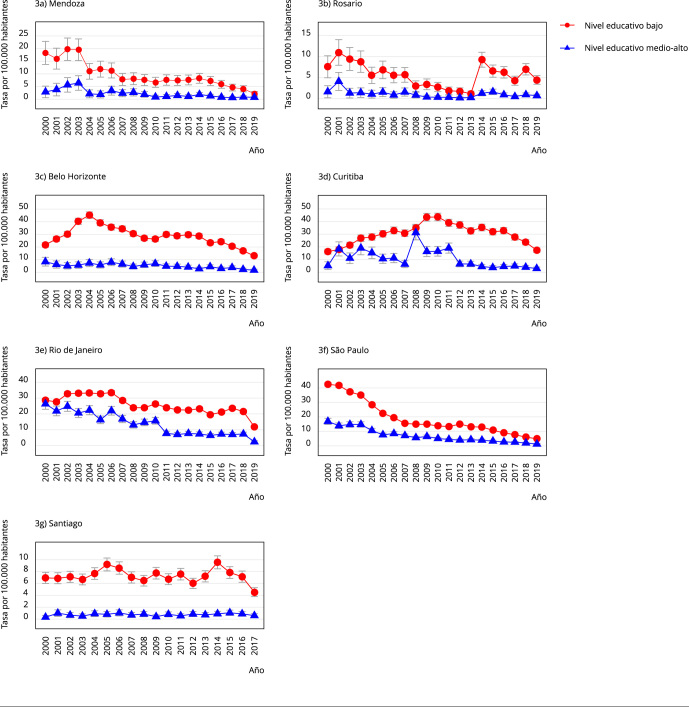
Tasa anual de homicidio según nivel educativo en siete ciudades de Sudamérica, 2000-2019.

**Figura 4:**
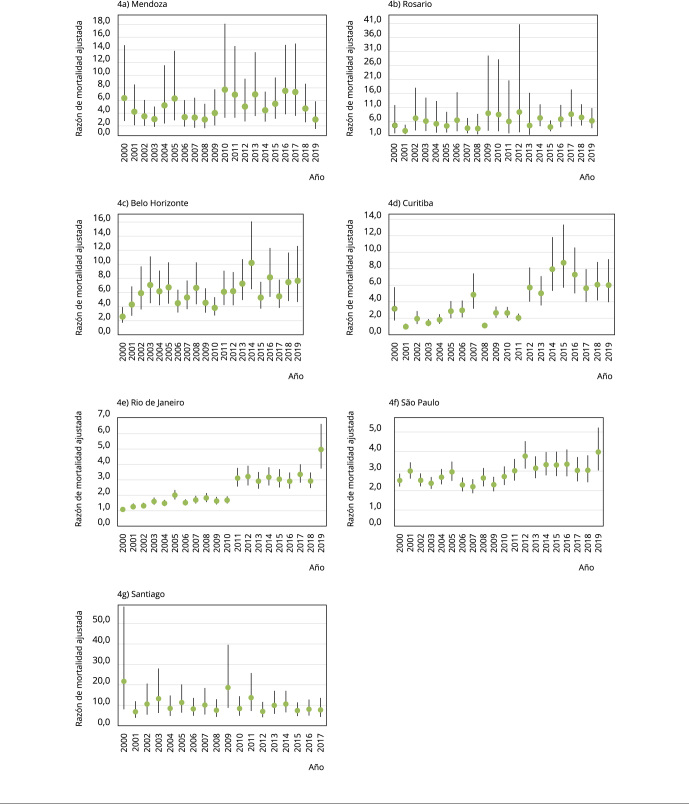
Razones de mortalidad anual, tomando como referencia la mortalidad por homicidio en población de nivel educativo medio-alto, en siete ciudades de Sudamérica, 2000-2019.

Los resultados, considerando solo homicidios como variable de respuesta, evidentemente fueron muy similares a los obtenidos considerando homicidios más muertes por lesión de intención no determinada. La desigualdad relativa educacional tuvo valores similares considerando solo homicidios, comparada a homicidios más muertes por lesión de intención no determinada (Tabla 4), excepto en Rosario y Belo Horizonte, con riesgos relativos más altos considerando solo homicidios. A excepción de Rosario, las variaciones temporales de la mortalidad educacional por homicidio no mostraron diferencias grandes entre homicidios, incluyendo muertes por lesión de intención no determinada y homicidios sin incluir estas muertes (Figura 3). Lo mismo puede observarse en el caso de las desigualdades relativas (Figura 4). Con respecto a las desigualdades educacionales de homicidio entre años de recesión y crecimiento económico, se obtuvieron resultados similares (Tabla 7), a excepción de São Paulo que, al considerar solo homicidios, no tuvo una diferencia estadísticamente significativa, aunque cercana (valor de p = 0,06), en las desigualdades relativas educacionales de homicidio entre ambos tipos de años (Tabla 8).

Nota: las barras verticales son intervalos de 95% de confianza (IC95%). Tasas de mortalidad estimadas, incluyendo todas las variables explicativas y una interacción entre el año y el nivel educativo. La variable dependiente son conteos agregados de homicidios (X85-X99).

Nota: las barras verticales son intervalos de 95% de confianza (IC95%) para las razones de tasas de mortalidad calculadas con la mortalidad, incluidas todas las variables explicativas y una interacción entre el año y el nivel educativo. La variable dependiente son conteos agregados de homicidios (X85-X99).

**Tabla 7:** Tasas de homicidio ajustadas y riesgos relativos (RR) entre grupos educativos (desigualdad relativa) en años de recesión *versus* años de expansión, 2000-2015.

País/Ciudad	Nivel educativo	Tasas de homicidio (IC95%)		RR (IC95%)	
		Expansión	Recesión	Expansión	Recesión
Argentina					
Mendoza	Medio-alto	0,88 (0,59-1,18)	0,89 (0,40-1,37)	Referencia	Referencia
	Bajo	7,69 (7,10-8,28)	10,01 (8,94-11,08)	8,72 (6,20-12,25)	11,30 (6,49-19,66)
Rosario	Medio-alto	0,27 (0,12-0,42)	0,34 (0,07-0,61)	Referencia	Referencia
	Bajo	3,61 (3,27-3,95)	5,93 (5,22-6,63)	13,13 (7,56-22,80)	17,36 (7,73-39,00)
Brasil					
Belo Horizonte	Medio-alto	5,80 (5,16-6,43)	4,23 (3,30-5,16)	Referencia	Referencia
	Bajo	32,13 (31,47-32,80)	26,26 (25,19-27,33)	5,54 (4,96-6,19)	6,21 (4,97-7,76)
Curitiba	Medio-alto	13,78 (12,63-14,92)	7,78 (6,50-90,5)	Referencia	Referencia
	Bajo	30,75 (29,90-31,59)	35,43 (34,02-36,83)	2,23 (2,05-2,43)	4,56 (3,85-5,39)
Rio de Janeiro	Medio-alto	15,20 (14,54-15,87)	12,55 (11,69-13,40)	Referencia	Referencia
	Bajo	27,90 (27,51-28,28)	25,23 (24,71-25,74)	1,84 (1,75-1,92)	2,01 (1,87-2,16)
São Paulo	Medio-alto	6,56 (6,23-6,89)	7,69 (7,23-8,14)	Referencia	Referencia
	Bajo	19,34 (19,07-19,60)	24,45 (24,07-24,83)	2,95 (2,80-3,10)	3,18 (2,99-3,38)
Chile					
Santiago	Medio-alto	0,80 (0,68-0,91)	0,42 (0,11-0,72)	Referencia	Referencia
	Bajo	7,44 (7,17-7,71)	7,74 (6,75-8,73)	9,34 (8,08-10,80)	18,60 (8,77-39,45)
Ciudades de Brasil *	Medio-alto	10,31 (10,00-10,62)	14,58 (13,89-15,28)	Referencia	Referencia
	Bajo	25,38 (25,10-25,65)	39,00 (38,08-39,93)	2,46 (2,39-2,54)	2,67 (2,56-2,79)
Todas las ciudades *	Medio-alto	6,22 (6,04-6,41)	8,78 (8,37-9,19)	Referencia	Referencia
	Bajo	17,33 (17,14-17,52)	24,70 (24,16-25,25)	2,78 (2,70-2,87)	2,81 (2,69-2,94)

IC95%: intervalos de 95% de confianza.

Nota: todas las estimaciones resultan de modelos de Poisson, cuya variable dependiente son conteos de homicidios (X85-X99), ajustando por sexo y edad.

* Ambos modelos además incluyen año y ciudad como efectos fijos.

**Tabla 8:** Cociente entre riesgos relativos (RR) de desigualdad educativa de homicidio, tomando la desigualdad educativa de homicidio en años de expansión económica como referencia, 2000-2015.

País/Ciudad	Tipo de año	**Cociente de desigualdades educativas entre años de recesión *versus* expansión**
Argentina		
Mendoza	Expansión	Referencia
	Recesión	1,30 (0,68-2,48)
Rosario	Expansión	Referencia
	Recesión	1,32 (0,50-3,52)
Brasil		
Belo Horizonte	Expansión	Referencia
	Recesión	1,12 (0,87-1,44)
Curitiba	Expansión	Referencia
	Recesión	2,04 (1,69-2,46) *
Rio de Janeiro	Expansión	Referencia
	Recesión	1,10 (1,01-1,19) **
São Paulo	Expansión	Referencia
	Recesión	1,08 (1,00-1,17) ***
Chile		
Santiago	Expansión	Referencia
	Recesión	1,99 (0,93-4,28) ***
Ciudades de Brasil ^#^	Expansión	Referencia
	Recesión	1,09 (1,03-1,15) **
Todas las ciudades ^#^	Expansión	Referencia
	Recesión	1,01 (0,96-1,06)

IC95%: intervalos de 95% de confianza.

Nota: todas las estimaciones resultan de modelos de Poisson, cuya variable dependiente son conteos de homicidios (X85-X99), ajustando por sexo y edad.

* Valor de p < 0,001;

** Valor de p < 0,05;

*** Valor de p < 0,10;

^#^ Ambos modelos además incluyen año y ciudad como efectos fijos.

## Discusión

Los hallazgos principales de este estudio son: (1) hubo diferencias marcadas en la evolución temporal de las tasas de homicidio entre las siete ciudades; (2) todas las ciudades tuvieron desigualdades educacionales en el riesgo de homicidio, siendo siempre las poblaciones de nivel educativo bajo las más vulnerables; (3) hubo diferencias en la variación temporal de estas desigualdades, aunque en ninguna ciudad se observó disminución de las desigualdades educacionales por homicidio durante los últimos años del periodo 2000-2019; (4) las cuatro ciudades de Brasil, analizadas en conjunto, tuvieron desigualdades educativas relativas de homicidio mayores en años de recesión económica, con respecto a años de crecimiento económico.

El análisis general realizado en todas las ciudades parece indicar una mayor desigualdad educacional de la mortalidad por homicidios durante los años de recesión económica, y esta desigualdad tuvo riesgos relativos significativamente mayores en Curitiba, Rio de Janeiro, São Paulo, y en el conjunto de las cuatro ciudades brasileras. En el caso de Rio de Janeiro, la fragmentación de los grupos criminales en un contexto de crisis económica podría haber llevado a mayores disputas por el control de territorios, incrementando la frecuencia de homicidios tanto entre miembros de grupos criminales como también población no involucrada en el conflicto. En este sentido, se ha planteado que el buen funcionamiento del mercado de drogas ilegales en Rio de Janeiro se asocia a ausencia de disputas por nuevos territorios ^11^. Debido a la localización concentrada de estos enfrentamientos en *favelas* y áreas adyacentes ^35^, es probable que un gran porcentaje de estas víctimas corresponda a población de nivel educativo bajo, incrementando los niveles de desigualdad educativa por homicidio.

En Argentina, tanto en Mendoza como en Rosario, no parece haber asociación entre crisis económica y aumento de las desigualdades educativas del homicidio. En Rosario parece existir un contexto de alta fragmentación criminal que, asociado a una expansión del consumo de drogas ilegales -especialmente cocaína-, podría incrementar los niveles de violencia letal producto de la disputa por nuevos mercados de consumo ^36^. Si este aumento de consumo podría estar asociado a años de crecimiento económico ^37^, sería esperable un aumento de las desigualdades sociales de homicidio en Rosario. Sin embargo, no parece claro por qué estos niveles de violencia tendrían que disminuir con una retracción del consumo de drogas ilegales en un contexto de crisis económica.

Las variaciones temporales de las tasas de homicidio en ambos niveles educativos parecen dar más apoyo a la hipótesis institucionalista, particularmente en las ciudades de Brasil. En Belo Horizonte, el programa *Fica Vivo* (Quedarse Vivo), implementado mayormente a partir de 2005 en áreas de concentración geográfica alta de homicidios, estuvo asociado a la disminución de homicidios a nivel de ciudad en años posteriores ^38^ ^,^ ^39^. Con respecto a Curitiba, el descenso abrupto de la tasa de homicidio en población de nivel educativo bajo-medio a partir de 2014 pudo deberse a la implementación de políticas de seguridad pública a partir de 2012, especialmente en barrios de nivel socioeconómico bajo de Curitiba ^40^. Similarmente, en Rio de Janeiro se observó un descenso de las tasas de homicidio en ambos niveles educativos a partir de 2009, en coincidencia con la implementación del Programa de Seguridad Pública, iniciado el mismo año ^41^. Este programa consistió en el establecimiento de policías comunitarias en *favelas* con el objetivo de aumentar el control estatal de dichas áreas ^40^. En cambio, en São Paulo pareció predominar un proceso de monopolización del crimen organizado detrás de la baja de la tasa de homicidios ^42^ ^,^ ^43^ ^,^ ^44^. Las caídas más abruptas de las tasas de homicidio en ambos niveles educativos coinciden con la expansión hegemónica del Primer Comando Capital - una organización criminal surgida en cárceles del Estado de São Paulo - en la periferia de São Paulo durante 2001-2006 ^45^. Durante el mismo periodo hubo un aumento constante de la tasa de encarcelamiento asociado a una posterior disminución de la tasa de homicidio ^46^.

A pesar del descenso de las tasas de homicidio en la segunda mitad del periodo 2000-2019 en Curitiba, Rio de Janeiro y São Paulo, pareció haber un aumento de las desigualdades relativas educacionales de homicidio en estas ciudades durante los últimos años del periodo. Este proceso podría ser indicativo de una creciente concentración geográfica de homicidios en áreas de violencia persistente. Tomando el caso de São Paulo, el periodo de descenso de las tasas de homicidio coincide con una disminución en la superficie geográfica de conglomerados de mortalidad alta ^47^, aunque todavía concentrada en áreas de violencia endémica -áreas con permanencia temporal de tasas altas de homicidio. En paralelo, se observó un incremento en el porcentaje de personas fallecidas producto de la confrontación con fuerzas de seguridad ^47^. El mismo fenómeno parece darse en Rio de Janeiro donde, en paralelo a un aumento de este tipo de muertes ^48^, se ha observado un incremento de personas desaparecidas en áreas con frecuencia alta de homicidios ^49^.

No todos los homicidios tienen algún grado de involucramiento con el mercado de drogas ilegales o el crimen organizado y pueden experimentar variaciones asociadas a los ciclos económicos. Eventos estresantes a nivel macro-social aparejados a las crisis económicas, como el desempleo, las quiebras de empresas, el aumento de la pobreza, pueden desencadenar hechos violentos cuyo desenlace podría ser la agresión letal. Prueba de ello es el siguiente relato extraído de un libro reciente sobre pobreza urbana en Argentina: “*estaba sin laburo* [trabajo]*, no tenía para comer, mi hijo no tenía pañales y agarré y le dije a un amigo que vayamos a robar*” ^50^ (p. 136). Es así como las crisis económicas pueden ser vistas como parte de un gradiente de violencia, de arriba hacia abajo, interactuando con otros encadenamientos de violencia a nivel comunitario y familiar ^50^. Desde esta perspectiva, tanto la violencia asociada a mercados ilícitos como la no asociada a este mercado podrían estar conectadas en ocasiones. En el caso de las ciudades brasileras, se ha estimado que un 50% y 45% de los homicidios en Belo Horizonte y Maceió estuvieron relacionados, directa o indirectamente, con el mercado de drogas ilícitas, respectivamente ^10^. Similarmente, en Argentina, se ha estimado un 40% de homicidios vinculados al narcotráfico ^51^.

Este estudio presenta varias limitaciones. Primero, aunque la fusión de muertes por lesión de intención no determinada y homicidios podría incorporar otros tipos de lesiones (suicidios y lesiones no intencionales) encubiertas como muertes por lesión de intención no determinada, es probable que un porcentaje significativo de las muertes por lesión de intención no determinada corresponda a homicidios. Salvo Rosario, las restantes ciudades no mostraron diferencias importantes en las variaciones temporales de las tasas de homicidio por nivel educativo. De manera similar a lo encontrado en Argentina, utilizando una técnica de imputación de muertes por lesión de intención no determinada al resto de las muertes por lesiones ^26^, la combinación de muertes por lesión de intención no determinada con homicidios mostró variaciones temporales similares comparadas a los homicidios, pero en niveles más altos de mortalidad. Con respecto a Rosario, el descenso abrupto de la tasa de homicidio durante 2008-2013 no parece coincidir con las investigaciones que dan cuenta de un incremento de la violencia letal durante ese período, a partir de disputas territoriales entre las dos bandas criminales más importantes de la ciudad ^36^. Segundo, el uso de proyecciones poblacionales lineales no considera variaciones no-lineales de las poblaciones como consecuencia de cambios macroeconómicos u otros procesos socioculturales. Tercero, no fue posible analizar las variaciones temporales en las tasas de homicidios por sexo y nivel educativo debido al bajo número de muertes de mujeres, especialmente en Mendoza, Rosario y Santiago. Cuarto, el aumento de las desigualdades relativas educativas por homicidio en Curitiba, Rio de Janeiro y São Paulo coincide con la mejora en el registro de nivel educativo en homicidios y muertes por lesión de intención no determinada hacia los últimos años del periodo 2000-2019. Los coeficientes de correlación de Pearson entre los riesgos relativos de desigualdad por homicidio más muertes por lesión de intención no determinada (Figura 2) y el porcentaje de estas muertes con dato de nivel educativo (Material Suplementario - Tabla S1; https://cadernos.ensp.fiocruz.br/static//arquivo/supl-e00228923_8967.pdf) fueron 0,56, 0,65 y 0,80 en Curitiba, Rio de Janeiro y São Paulo, respectivamente. Esto podría indicar que la mejora en el registro de nivel educativo sería mayor en muertes con nivel educativo bajo, aumentado artificialmente las desigualdades relativas educativas por homicidio en los últimos años de 2000-2019. Sin embargo, no tenemos conocimiento acerca de estudios que evalúen la sobreestimación o subregistro de información sobre nivel educativo en estas ciudades.

Por un lado, la “guerra urbana” ^11^ contra las drogas, entendida como el uso de la fuerza indiscriminado por parte del Estado enfocado en grupos criminales situados en barrios de nivel socioeconómico bajo, parece haber llevado a una creciente desigualdad social de la mortalidad por homicidio. Por el otro, en un contexto de fragmentación criminal y crisis económica se podrían agravar estas desigualdades a través de mayores disputas territoriales en estas áreas.
