# Supplementary material for: Homicides in southern South American cities: educational inequalities and economic fluctuations
Source: Cad Saude Publica. 2024 Jun 21;40(6):e00228923. [Article in Spanish] doi: 10.1590/0102-311XES228923 (PMC11192574; doi:10.1590/0102-311XES228923)
Supplement: Supplementary file 1 [file 1678-4464-csp-40-06-ES228923-s.pdf]

Cuadro S1 Municipios metropolitanos incluidos y excluidos del análisis en cuatro ciudades de Brasil.

CIUDAD	MUNICIPIOS INCLUIDOS	MUNICIPIOS EXCLUIDOS *
Belo Horizonte	Belo Horizonte, Betim, Brumadinho, Caeté, Contagem, Ibirité, Igarapé, Matozinhos, Pedro Leopoldo, Ribeirão das Neves, Sabará, Santa Luzia, São Joaquim de Bicas	Capim Branco, Confins, Esmeraldas, Florestal, Itaguara, Itatiaiuçu, Jaboticatubas, Nova União, Juatuba, Lagoa Santa, Mario Campos, Mateus Leme, Nova Lima, Raposos, Rio Acima, Rio Manso
Curitiba	Almirante Tamandaré, Araucária, Campo Largo, Campo Magro, Colombo, Curitiba, Fazenda Rio Grande, Itaperuçu, Mandirituba, Pinhais, Piraquara, Rio Branco do Sul, Rio Negro, São José Dos Pinhais	Adrianópolis, Agudos do Sul, Balsa Nova, Bocaiuva do Sul, Campina Grande do Sul, Campo do Tenente, Cerro Azul, Contenda, Lapa, Piên, Quatro Barras, Quitandinha, Tijucas do Sul, Tunas do Paraná, Doutor Ulysses
Rio de Janeiro	Belford Roxo, Cachoeiras de Macacu, Duque de Caxias, Guapimirim, Itaboraí, Itaguaí, Japeri, Magé, Maricá, Mesquita, Nilópolis, Niterói, Nova Iguaçu, Queimados, Rio Bonito, Rio de Janeiro, São Gonçalo, São João de Meriti, Seropédica, Tanguá	Paracambi
São Paulo	Arujá, Barueri, Biritiba-Mirim, Caieiras, Cajamar, Carapicuíba, Cotia, Diadema, Embu das Artes, Embu-Guaçu, Ferraz de Vasconcelos, Francisco Morato, Franco da Rocha, Guararema, Guarulhos, Itapevi, Itaquaquecetuba, Jandira, Juquitiba, Mairiporã, Mauá, Mogi das Cruzes, Osasco, Poá, Ribeirão Pires, Rio Grande da Serra, Santa Isabel, Santana de Parnaíba, Santo André, São Bernardo do Campo, São Caetano do Sul, São Paulo, Suzano, Taboão da Serra, Vargem Grande Paulista	Itapecerica da Serra, Pirapora do Bom Jesus, Salesópolis, São Lourenço da Serra

Nota: el total de municipios de cada ciudad comprende una región metropolitana delimitada por el Instituto Brasileiro de Geografia e Estatística.
* Los municipios excluidos del análisis son aquellos que durante el censo de 2000 contaban con menos de 20.000 habitantes.

Tabla S1 Porcentaje de muertes (homicidios + MLIND -muerte por lesión de intención no determinada-Y10-Y34) con dato de nivel educativo en siete ciudades de Sudamérica, 2000-2019.

Año	Mendoza		Rosario		Belo Horizonte		Curitiba		Rio de Janeiro		São Paulo		Santiago	
	% con nivel educativo	Muertes totales	% con nivel educativo	Muertes totales	% con nivel educativo	Muertes totales	% con nivel educativo	Muertes totales	% con nivel educativo	Muertes totales	% con nivel educativo	Muertes totales	% con nivel educativo	Muertes totales
2000	96	73	76	49	94	833	81	488	74	3.929	85	7.221	100	315
2001	99	72	90	72	96	986	83	511	74	4.000	83	7.131	100	265
2002	99	97	86	64	98	982	94	525	78	4.342	83	7.381	100	230
2003	100	99	90	82	97	1.355	94	629	80	4.426	87	6.045	100	214
2004	95	60	95	62	98	1.535	96	596	87	3.927	87	5.389	100	275
2005	98	65	84	100	97	1.428	91	665	88	4.180	86	4.665	100	297
2006	96	78	95	105	97	1.410	91	747	90	4.053	79	4.127	100	282
2007	100	53	98	113	96	1.384	94	712	90	4.436	83	3.548	100	228
2008	100	60	95	187	96	1.262	94	890	92	4.121	83	3.384	100	214
2009	97	65	92	179	96	1.293	94	1.027	95	4.323	84	3.500	99	248
2010	97	58	95	320	98	1.136	98	995	94	3.361	88	3.235	100	222
2011	98	62	94	286	97	1.407	99	995	96	3.258	91	3.151	100	244
2012	99	71	91	234	97	1.166	99	919	96	3.061	91	3.155	100	202
2013	98	80	87	296	96	1.279	99	853	97	3.169	91	2.924	100	237
2014	100	101	79	280	96	1.250	98	928	96	2.702	92	2.938	100	314
2015	97	97	82	233	96	1.064	98	854	91	2.565	93	2.575	100	265
2016	99	95	86	259	96	1.166	99	822	92	2.957	93	2.360	100	242
2017	99	82	92	228	97	1.099	98	731	92	3.232	93	2.510	100	154
2018	96	85	88	288	95	920	99	619	93	3.097	93	3.464	ND	ND
2019	85	111	90	227	97	932	99	521	93	4.049	94	3.126	ND	ND

ND: datos no disponibles.
